# Joint-level proprioceptive deficits and postural instability in Fibromyalgia: a biomechanical assessment using digital inclinometry and dynamic posturography 

**DOI:** 10.3389/fbioe.2025.1622679

**Published:** 2025-09-10

**Authors:** Praveen Kumar Kandakurti, Ravi Shankar Reddy, Hani Hassan Alnakhli, Batool A. Alkhamis, Ghada M. Koura, Mohammad A. ALMohiza, Faisal M. Alyazedi, Debjani Mukherjee, Vikram Sreenivasa Rao

**Affiliations:** ^1^ College of Health Sciences, Gulf Medical University, Ajman, United Arab Emirates; ^2^ Physical Therapy Program, Department of Medical Rehabilitation Sciences, College of Applied Medical Sciences, King Khalid University, Abha, Saudi Arabia; ^3^ Department of Health Rehabilitation Sciences, College of Applied Medical Sciences, King Saud University, Riyadh, Saudi Arabia; ^4^ Physical Therapy Department, Prince Sultan Military College of Health Sciences, Dahran, Saudi Arabia; ^5^ Department of Anatomy, College of Medicine, King Khalid University, Abha, Saudi Arabia

**Keywords:** Fibromyalgia, proprioception, postural balance, joint position sense, posturography

## Abstract

**Objective:**

Fibromyalgia syndrome (FMS) is characterized by chronic musculoskeletal pain, fatigue, and sensory disturbances, often leading to impaired proprioception and postural control. This study aimed to examine joint reposition sense (JRS) at the hip, knee, and ankle, alongside limits of stability (LOS), in elderly individuals with FMS using digital inclinometers and computerized posturography.

**Methods:**

A total of 108 participants (54 with FMS, 54 age-matched healthy controls) were assessed. JRS was assessed at standardized joint angles of the hip (60° flexion), knee (45° flexion), and ankle (15° plantarflexion) using calibrated digital inclinometers, while LOS parameters—reaction time, maximum excursion, and directional control—were recorded with dynamic posturography.

**Results:**

Participants with FMS showed significantly higher joint position errors at the hip (mean difference = 2.53°), knee (2.51°), and ankle (2.24°) (p < 0.001, Cohen’s d > 1.8). LOS parameters were also impaired in the FMS group, with slower reaction time (Δ = 0.97 s), reduced maximum excursion (Δ = −3.44%), and lower directional control (Δ = −22.64%) (all p < 0.001). JRS errors negatively correlated with LOS metrics, particularly at the hip and knee. Regression analysis confirmed JRS as a significant predictor of postural control.

**Conclusion:**

Lower limb proprioceptive deficits significantly impact postural stability in individuals with FMS. Targeted proprioceptive training, especially at the hip and knee, may improve functional balance and reduce fall risk in this population.

## Background

Fibromyalgia syndrome (FMS) is a perplexing and debilitating chronic pain condition that affects millions of individuals worldwide, characterized predominantly by widespread musculoskeletal pain, fatigue, and sleep disturbances (Asiri et al., 2023). However, the clinical picture of FMS is not limited solely to these cardinal symptoms; it includes a diverse range of physical and functional impairments that can severely impact an individual’s quality of life (Scarano et al., 2022). Among these, compromised joint reposition sense (JRS), postural control, and functional balance have garnered attention as areas of particular concern for individuals living with FMS (Scarano et al., 2022). This study seeks to unravel the intricate interplay between these impairments and the severity of FMS symptoms, offering valuable insights that can inform research and clinical practice (Ong et al., 2022).

Proprioception, often referred to as our “sixth sense,” is a crucial sensory ability that enables us to perceive and understand the position, movement, and orientation of our body parts in space without relying on visual cues (Asiri et al., 2023). Central to this proprioceptive capacity is JRS, which specifically pertains to our ability to accurately perceive the relative positions and movements of our joints, such as those in the hip, knee, and ankle (Scarano et al., 2022). This sensory information is gathered through specialized receptors known as proprioceptors, primarily located in the muscles, tendons, and ligaments surrounding our joints (Scarano et al., 2022). JRS plays a fundamental role in coordinating our movements, maintaining balance, and preventing injury by providing real-time feedback to our central nervous system, allowing for precise control of muscle contractions and joint positions (Ong et al., 2022). Dysfunction in proprioception and JRS can have significant implications for individuals, particularly in conditions like FMS, where impairments in these sensory processes may contribute to altered movement patterns and increased susceptibility to musculoskeletal issues (Alshahrani and Reddy, 2023; Raizah et al., 2023a; Raizah et al., 2023b). Understanding these sensory mechanisms is vital for clinical evaluation and for designing effective rehabilitation strategies and interventions to enhance proprioceptive function and overall physical wellbeing (Alshahrani and Reddy, 2023; Raizah et al., 2023a; Raizah et al., 2023b).

Limits of stability (LOS) refers to the maximum range of movement or displacement of the body’s center of mass that an individual can achieve while maintaining balance and stability without falling (Asiri et al., 2023). This concept is critical for understanding human postural control and mobility (Asiri et al., 2023). To determine one’s LOS, the nervous system constantly processes sensory information from the visual, vestibular, and proprioceptive systems and calculates the potential balance boundaries (Asiri et al., 2023). These boundaries are dynamic and can vary among individuals and situations. Expanding one’s LOS is essential for daily living activities and preventing falls, as it enables individuals to reach, step, and react effectively while maintaining equilibrium, thus emphasizing the importance of balance training and rehabilitation programs in promoting overall mobility and safety (Asiri et al., 2023).

Comparing JRS and LOS between individuals with FMS and healthy controls is essential for gaining insights into the impact of this condition on proprioception and balance. This investigation plays a critical role in developing personalized interventions, enhancing the quality of life for FMS patients, and advancing our clinical understanding of the syndrome (Asiri et al., 2023). By carefully examining the proprioceptive abilities and balance thresholds of these two distinct groups, this research aims to reveal significant differences that illuminate how FMS affects sensory and motor function (Asiri et al., 2023). These insights have the potential to inform the development of targeted therapies, alleviating balance impairments and enhancing the wellbeing of individuals with FMS. This study promises to deepen our understanding of the complex nature of FMS’s impact on musculoskeletal function, thus advancing rehabilitation practices and offering improved patient care.

For several reasons, exploring the potential correlations between JRS and LOS in the hip, knee, and ankle among individuals with FMS holds significant importance. Firstly, understanding how these proprioceptive and balance-related factors interrelate can provide valuable insights into the underlying mechanisms of balance disturbances in FMS patients (Asiri et al., 2023). Secondly, identifying correlations may help pinpoint specific areas of impairment within the musculoskeletal system, guiding the development of targeted rehabilitation strategies (Scarano et al., 2022). Moreover, establishing connections between JRS and LOS can contribute to a more comprehensive clinical evaluation of FMS, enhancing our ability to assess and manage balance issues in affected individuals (Scarano et al., 2022). Ultimately, this research has the potential to bridge the gap in our understanding of the intricate relationship between proprioception and balance in FMS, with potential implications for the development of tailored interventions to improve these patients’ daily functioning and wellbeing (Ong et al., 2022). This research aims to fill gaps in our understanding through two primary objectives. First, it seeks to conduct a comprehensive comparative analysis of JRS and LOS in the hip, knee, and ankle among individuals diagnosed with FMS compared to a healthy control group. Second, the study investigates potential correlations between JRS and LOS in the hip, knee, and ankle among individuals with FMS.

## Methods

### Design

The research utilized a comparative cross-sectional study design from June 2022 to August 2023 to investigate JRS and stability limits in individuals with FMS compared to a healthy control group. Ethical considerations were a paramount aspect of this study’s framework. Approval was obtained from the Institutional Review Board committee (DSR, KKU–REC# 334-098–45), confirming adherence to the stringent ethical standards delineated in the Declaration of Helsinki.

### Settings

The study was conducted in a clinical physical therapy biomechanics lab at DMRS, CAMS, KKU, and Aseeer, Saudi Arabia. Clinical evaluations and assessments occur in a dedicated healthcare facility or clinic equipped with the necessary tools for clinical examinations. Laboratory-based assessments, including advanced balance testing, took place in a controlled research laboratory outfitted with force platforms and specialized equipment to ensure precise and standardized measurements.

### Participants

Participants were selected based on specific inclusion and exclusion criteria. Participants were recruited consecutively from patients attending the outpatient rheumatology and physical therapy clinics at King Khalid University Hospital and affiliated community health centers between June 2022 and August 2023. Potential participants with a prior diagnosis of FMS were identified from clinical records and screened in person by a trained research physiotherapist. Screening involved a two-stage process: (1) verification of FMS diagnosis according to the 2016 revised ACR criteria through medical record review and clinical examination, and (2) confirmation of eligibility based on inclusion and exclusion criteria through structured interviews and physical assessment. Age-matched healthy controls were recruited from hospital staff, patient relatives, and community volunteers via posters and word-of-mouth, applying the same exclusion criteria. All eligible individuals received verbal and written explanations of the study protocol, potential risks, and benefits before providing written informed consent in accordance with institutional ethics approval. The inclusion criteria included individuals who had previously been diagnosed with FMS, meeting the ACR criteria for an FMS diagnosis (Alshahrani and Reddy, 2023), and age-matched healthy individuals without a history of FMS or chronic pain conditions, all 18 years or older. The diagnosis of FMS in a patient depended on meeting three key conditions. Firstly, the patient needed to have achieved a Widespread Pain Index (WPI) score of at least 7, indicating the presence of widespread pain throughout the body (Raizah et al., 2023a). Furthermore, their Symptom Severity (SS) Scale Score should have been five or higher, reflecting the severity of associated symptoms (Raizah et al., 2023a). These symptomatology indicators encompass widespread pain and other characteristic FMS symptoms and were required to have persisted consistently for a minimum of 3 months. Exclusion criteria included any history of vestibular disorders, significant lower limb injury or surgery within the past year, diagnosed peripheral neuropathy, uncontrolled cardiovascular conditions, or any medical or psychiatric condition deemed by the investigator to interfere with safe participation or accurate assessment of proprioception and postural stability.

The inclusion criteria for the healthy control group encompassed individuals aged 18 years or older with no history of FMS or chronic pain conditions, possessing adequate cognitive abilities to understand and follow test instructions, and being free from acute medical conditions that could hinder participation in physical assessments. In contrast, the exclusion criteria for this group involved individuals under the age of 18, those with a history or current diagnosis of FMS or chronic pain conditions, the presence of neurological or musculoskeletal disorders that could significantly affect proprioception or balance, severe cognitive impairments impeding the ability to comprehend test instructions, the existence of acute medical conditions precluding participation in physical assessments, and unwillingness to provide informed consent or participate voluntarily in the study.

### Lower extremity proprioception assessment

Digital inclinometers (Model: Dualer IQ Pro, JTECH Medical) were utilized for JRS assessment. Calibration was performed daily before testing sessions, following manufacturer-recommended procedures. Devices were zeroed on a flat, horizontal surface before each use to ensure accuracy. Measurement error was controlled by checking the inclinometer’s reference angle before each participant’s trial. The intra-rater reliability of the device has been previously validated with an ICC > 0.90 in lower limb assessments. This study outlined a comprehensive evaluation protocol to assess proprioceptive accuracy in individuals with FMS, focusing on the hip, knee, and ankle joints. We conducted these assessments using digital inclinometers, ensuring precise and consistent joint angle measurements. The assessments took place in a controlled, distraction-free environment to optimize the accuracy of proprioceptive feedback. Participants were blindfolded to eliminate visual cues, focusing solely on proprioceptive inputs. Participants lie supine for the hip JRS assessment, positioning the hip at 60 degrees of flexion. The inclinometer was securely fastened at the center of the thigh using a hook-and-loop strap. After maintaining the target position for 5 seconds, participants actively attempted to replicate it, verbally confirming their perceived accuracy. The knee JRS assessment involved participants seated with the knee initially flexed to 90°. The inclinometer was attached to the lateral aspect of the knee, and participants guided their knees to a 45-degree angle. After mentally noting this position, they tried to replicate it from the starting position, confirming alignment verbally. For the ankle, the assessment targeted a 15-degree plantar flexion. An inclinometer was affixed to the lateral side of the tibia and the outer edge of the foot. After positioning the ankle, participants were instructed to remember and replicate this angle, verbally stating their success. Each joint assessment was repeated three times to ensure reliability, with examiners providing consistent instructions throughout to standardize the testing conditions. All participants underwent testing in a fixed sequence to ensure procedural consistency: the hip JRS assessment was conducted first, followed by the knee JRS, ankle JRS, and finally the LOS assessment. A standardized rest interval of 2 minutes was provided between each joint repositioning task to minimize fatigue and learning effects. Before commencing the LOS assessment, participants were given a 5-min seated rest to further reduce fatigue-related variability. To control for examiner bias, all JRS assessments were performed by the same trained examiner, while a separate examiner, blinded to group allocation, conducted the LOS tests.

### Limits of stability assessment

**FIGURE 1:**
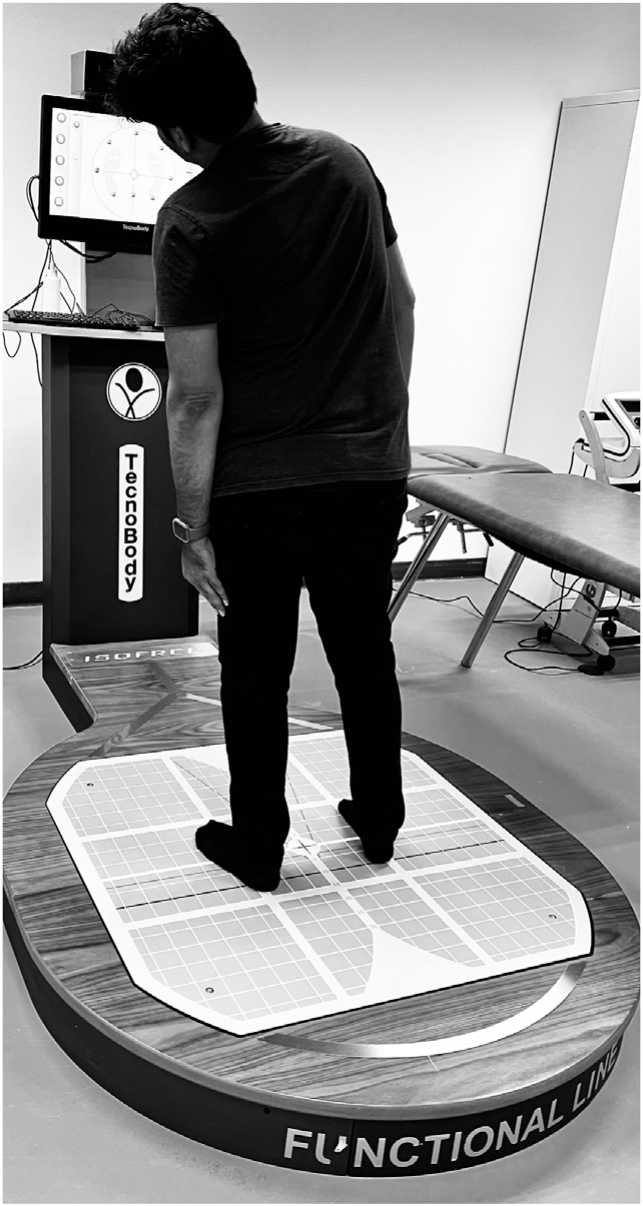
Limits of stability assessment using computerized stabilometric force platform.

The assessment of the Limits of Stability (LOS) in modern research utilizes dynamic posturography, incorporating advanced iso-free technology. Calibration was performed at the start of each testing day according to the manufacturer’s specifications. The force plate was zeroed with no load, and all sensors were tested for responsiveness using standard calibration weights. Measurement accuracy was verified with a maximum allowable error range of ±0.1° for angular displacement and ±0.5% for excursion measures. During the assessment, participants stand on a posturography platform with their feet positioned closely together while a display presents targets to guide their movements. The primary aim is to measure LOS by instructing individuals to shift their center of mass toward a designated target without changing their foot placement, covering eight distinct directions. The device meticulously records the extent of sway required to reach the target and assigns a performance score accordingly (Figure 1).

LOS assessments play a crucial role in evaluating an individual’s postural stability and control under various conditions. In this study, LOS is assessed through three key parameters: reaction time, maximum excursion, and directional control. Reaction time reflects the speed at which a person responds to unexpected disturbances or balance challenges, indicating their ability to adapt to sudden movements or uneven surfaces. Maximum excursion represents the furthest point an individual can lean or shift their weight in different directions while maintaining stability, providing insights into their range of motion and equilibrium control. Lastly, directional control evaluates a person’s capacity to maintain postural stability while adjusting their weight distribution along different axes—an essential skill for tasks that require quick directional changes, such as sports and everyday activities. To ensure reliability, all assessors underwent a 2-week training period. Intra-rater and inter-rater reliability checks were conducted on five pilot participants before main data collection, yielding ICC values of 0.88–0.93 for JRS and 0.91–0.95 for LOS parameters. All assessments occurred in the same lab environment under controlled lighting and noise conditions to minimize external variability.

### Data analysis

The normality of the data was verified using the Shapiro-Wilk test, which supported the use of parametric statistical methods by confirming a normal distribution (p > 0.05). Data analysis was conducted using SPSS version 24, employing independent t-tests to compare JRS and LOS between the FMS group and healthy controls. Results, expressed as mean values and standard deviations, established statistical significance at the conventional alpha level of 0.05, highlighting significant differences in JRS and LOS between the groups. Pearson correlation coefficients were calculated to analyze the relationships between JRS and LOS parameters within the FM group, assessing the strength and direction of these associations. Additionally, multiple linear regression was utilized to determine if JRS parameters could predict LOS outcomes, further exploring the interaction between proprioceptive abilities and postural stability in individuals with FMS, with all significance levels set at 0.05. For regression analyses, 95% confidence intervals (CIs) were calculated for all beta coefficients to provide an estimate of the precision of effect size estimates. No formal adjustments for multiple comparisons were applied, as the analysis was guided by *a priori* hypotheses regarding the relationships between JRS and LOS measures.

## Results

The demographic characteristics of the study population revealed no significant differences in age or gender distribution between the FMS group and the healthy control group (Table 1). However, BMI and education level showed significant differences, with the FMS group having a higher BMI and a lower education level than the controls. Additionally, the FMS group reported an average duration of symptoms of over 3 years, with elevated scores in both the Widespread Pain Index (WPI) and the Symptom Severity (SS) scale.

**TABLE 1:** Demographic Characteristics of the study population.

Characteristic	FMS group (n = 54) (Mean ± SD)	Healthy control group (n = 54) (Mean ± SD)	p-value
Age (years)	65.3 ± 6.6	64.8 ± 8.8	0.756
Gender (Male/Female)	25/29 (46.3%/53.7%)	28/26 (51.8%/48.1%)	0.710
Education level (Years)	14.6 ± 2.5	16.8 ± 3.9	0.001
Duration of symptoms (months) (Mean ± SD)	36.3 ± 12.3	-	-
Body mass index (BMI)	25.63 ± 2.13	24.12 ± 1.98	<0.001
Widespread pain index (WPI) (Mean ± SD)	9.3 ± 2.8	-	-
Symptom severity (SS) scale score (Mean ± SD)	7.8 ± 2.7	-	-

FMS, fibromyalgia syndrome; SD, standard deviation; BMI, body mass index; WPI, widespread pain index; SS, scale, Symptom Severity scale.

Significant differences were observed in lower extremity JRS and postural stability measures between participants with Fibromyalgia Syndrome (FMS) and healthy controls (Table 2). The FMS group exhibited higher joint position errors, with mean differences of 2.53° for the hip, 2.51° for the knee, and 2.24° for the ankle, all showing large effect sizes (Cohen’s d > 1.80, p < 0.001). Regarding postural stability, FMS participants demonstrated slower reaction times (mean difference = 0.97 s, Cohen’s d = 3.53, p < 0.001), reduced maximum excursion (mean difference = −3.44%, Cohen’s d = −2.67, p < 0.001), and poorer direction control (mean difference = −22.64%, Cohen’s d = −3.48, p < 0.001), all indicating substantial impairments compared to healthy controls.

**TABLE 2:** Lower extremity joint position errors and LOS measures between FMS and healthy participants.

Variables	Variables	FMS participants (n = 54) (mean ± SD)	Healthy controls (n = 54) (mean ± SD)	Mean difference	Effect size. (Cohen’s d)	p-value
Lower extremity JRS variables	Hip JRS (°)	4.5 ± 1.7	2.0 ± 0.9	2.5	1.86	<0.001
	Knee JRS (°)	3.9 ± 1.8	1.5 ± 0.7	2.5	1.80	<0.001
	Ankle JRS (°)	3.3 ± 1.1	1.1 ± 1.3	2.2	1.87	<0.001
Postural stability variables	Reaction time (s)	1.86 ± 0.37	0.89 ± 0.12	0.97	3.53	<0.001
	Maximum excursion (%)	6.23 ± 1.23	9.67 ± 1.34	−3.44	−2.67	<0.001
	Direction control (%)	69.99 ± 8.88	92.63 ± 2.45	−22.64	−3.48	<0.001

FMS, fibromyalgia syndrome; JRS, joint reposition sense; CI, confidence interval.

**FIGURE 2:**
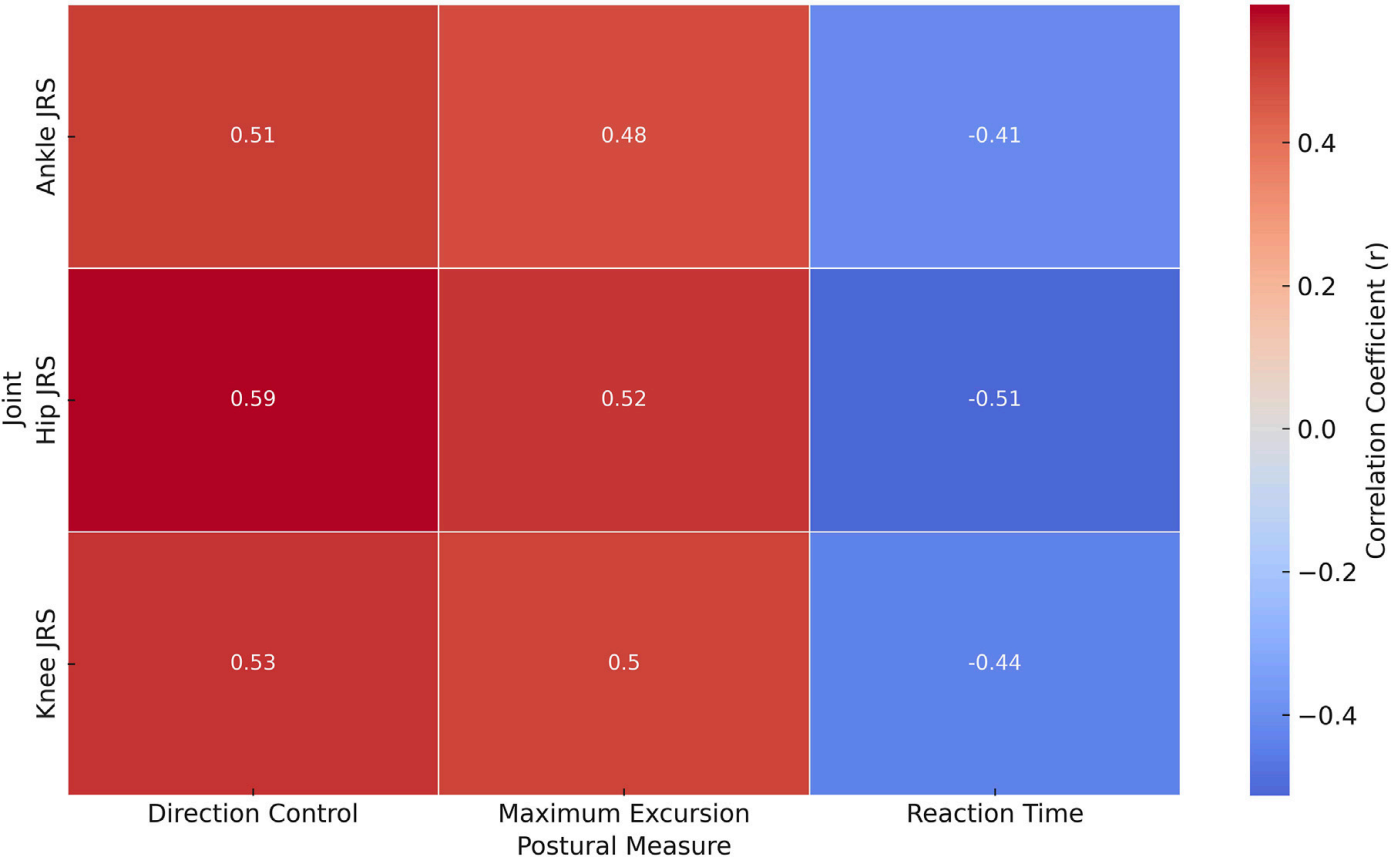
Heatmap of correlation coefficients between joint reposition sense and postural stability metrics in Fibromyalgia syndrome patients.

The results showed significant correlations between lower extremity JRS and various postural stability measures in individuals with FMS (Figure 2). Notably, Hip JRS showed a moderate negative correlation with reaction time (r = −0.513, p = 0.005) and positive correlations with maximum excursion (r = 0.524, p = 0.004) and direction control (r = 0.593, p = 0.006), suggesting that better hip joint positioning is associated with faster reaction times and improved control and range of postural movements. Similarly, Knee JRS and Ankle JRS were also negatively correlated with reaction time (Knee JRS: r = −0.437, p = 0.011; Ankle JRS: r = −0.413, p = 0.011) and positively correlated with maximum excursion and direction control, indicating a consistent pattern across joint senses affecting postural stability.

Multiple linear regression analysis revealed that JRS significantly predicted postural stability measures, with the hip, knee, and ankle JRS all showing strong associations with reaction time, maximum excursion, and direction control (Table 3). Hip JRS was a significant predictor for reaction time (β = 0.352, SE = 0.045, p < 0.001), maximum excursion (β = 0.268, SE = 0.036, p < 0.001), and direction control (β = 0.187, SE = 0.029, p < 0.001). Similarly, knee JRS showed even stronger associations with reaction time (β = 0.451, SE = 0.016, p < 0.001), maximum excursion (β = 0.378, SE = 0.022, p < 0.001), and direction control (β = 0.219, SE = 0.017, p < 0.001). Ankle JRS also significantly predicted reaction time (β = 0.120, SE = 0.020, p < 0.001), maximum excursion (β = 0.085, SE = 0.018, p = 0.003), and direction control (β = 0.098, SE = 0.026, p = 0.001). However, the effect sizes were smaller compared to the hip and knee.

**TABLE 3:** Multiple linear regression analysis results.

Predictor variable	Dependent variable	Beta coefficient (β)	Standard error (SE)	95% CI for β	p-value
Hip JRS (°)	Reaction time (s)	0.352	0.045	0.264 to 0.440	<0.001
	Maximum excursion (%)	0.268	0.036	0.197 to 0.339	<0.001
	Direction control (%)	0.187	0.029	0.130 to 0.244	<0.001
Knee JRS (°)	Reaction time (s)	0.451	0.016	0.420 to 0.482	<0.001
	Maximum excursion (%)	0.378	0.022	0.335 to 0.421	<0.001
	Direction control (%)	0.219	0.017	0.186 to 0.252	<0.001
Ankle JRS (°)	Reaction time (s)	0.120	0.020	0.081 to 0.159	<0.001
	Maximum excursion (%)	0.085	0.018	0.050 to 0.120	0.003
	Direction control (%)	0.098	0.026	0.047 to 0.149	0.001

JRS, joint reposition sense; β, Beta Coefficient; SE, standard error; CI, confidence intervals.

## Discussion

In this study, we aimed to achieve two primary objectives: firstly, to conduct a comprehensive comparative analysis of JRS and LOS in individuals with FMS and a healthy control group, discerning significant distinctions and their possible ramifications; and secondly, to investigate potential correlations between JRS and LOS in individuals afflicted with FMS. Our findings revealed that individuals with FMS exhibited notable deficits in JRS at the hip, knee, and ankle joints compared to healthy controls. Moreover, we identified significant correlations between JRS parameters and LOS outcomes within the FMS group, indicating that those with better proprioception exhibit improved postural stability. Multiple linear regression analysis further demonstrated the predictive potential of JRS parameters, particularly at the hip, for LOS outcomes in FMS individuals. These results collectively emphasize the importance of addressing proprioceptive deficits in FMS management and highlight the potential benefits of targeted interventions in enhancing postural stability and reducing the risk of falls.

The results revealed significant distinctions in proprioceptive abilities, with individuals in the FMS group exhibiting impaired JRS at the hip, knee, and ankle joints compared to the healthy control group. These findings align with previous research that has suggested proprioceptive deficits in FMS (Asiri et al., 2023; Alshahrani and Reddy, 2023; Raizah et al., 2023a; Raizah et al., 2023b; Reddy et al., 2022). One potential explanation for these results could be the widespread pain and sensory hypersensitivity often experienced by individuals with FMS (Palmer et al., 2019). These factors may disrupt the standard sensory feedback mechanisms crucial for accurate joint position perception (Palmer et al., 2019). Additionally, the chronic nature of FMS may lead to disuse and deconditioning, further contributing to proprioceptive impairments (D et al., 2020). Overall, these results underscore the multifaceted challenges individuals with FMS face in maintaining accurate JRS, which may affect their postural stability and overall quality of life (Asiri et al., 2023; Alshahrani and Reddy, 2023; Raizah et al., 2023a; Raizah et al., 2023b; Reddy et al., 2022). Several previous studies have supported the proprioceptive deficits observed in individuals with FMS. For instance, a survey by Gucmen et al. (2022) found that FMS patients displayed impaired JRS in the knee compared to healthy controls (Gucmen et al., 2022). Similarly, Ulus et al. (2013) reported deficits in proprioception among FMS patients, particularly in the lower extremities, suggesting that sensory processing abnormalities may contribute to these impairments (Ulus et al., 2013). Moreover, the study by Reddy et al. (2022) highlighted the presence of altered proprioception in FMS patients, emphasizing the role of central sensitization and sensory disturbances in this condition (Reddy et al., 2022). These collective findings from previous research support our results, indicating that individuals with FMS often exhibit compromised JRS, potentially due to the complex interplay of sensory and central processing mechanisms associated with this syndrome (Asiri et al., 2023; Alshahrani and Reddy, 2023; Raizah et al., 2023a; Raizah et al., 2023b; Reddy et al., 2022).

The impaired LOS observed in individuals with FMS compared to a healthy control group can be attributed to several interrelated factors (Jones et al., 2009). Firstly, FMS is characterized by widespread pain, fatigue, and musculoskeletal stiffness, which can hinder affected individuals’ normal range of motion and flexibility (Jones et al., 2009; Sempere-Rubio et al., 2018). These physical symptoms may lead to a reduced capacity to shift one’s body weight effectively, limiting their ability to reach the outer boundaries of their LOS (Rasouli et al., 2018). Secondly, FMS is associated with central sensitization, which can heighten the perception of pain and discomfort (Nielsen and Henriksson, 2007). This heightened sensory processing may make individuals with FMS more cautious in their movements, resulting in smaller excursions within their LOS to avoid potential discomfort or pain (Nielsen and Henriksson, 2007). Thirdly, the chronic nature of FMS often leads to physical deconditioning and reduced physical activity levels (Yunus, 2007; Rehm et al., 2021). As a result, individuals with FMS may experience muscle weakness and decreased muscle endurance, which can further compromise their ability to maintain postural stability and expand their LOS (Rehm et al., 2021). Numerous previous studies have reported impaired LOS in individuals with FMS compared to healthy control groups, corroborating the findings of our study. For example, research by Russek and Fulk. (2009) revealed that FMS patients exhibited reduced LOS in multiple directions, suggesting impaired postural control and stability. (Russek and Fulk, 2009). Similarly, a study conducted by Alshahrani and Reddy. (2023) found that FMS patients displayed compromised postural stability and reduced LOS compared to healthy individuals (Alshahrani and Reddy, 2023). Peinado-Rubia et al. (2023) reported limitations in LOS in FMS patients, particularly in the forward direction, indicating difficulties in maintaining balance and adapting to external perturbations (Peinado-Rubia et al., 2023). These collective findings underscore the consistent presence of impaired LOS in FMS individuals, highlighting the impact of this condition on postural control and stability, which can have significant implications for their daily activities and quality of life.

The results revealed significant correlations, indicating that individuals with FMS who demonstrated better proprioceptive abilities also exhibited improved postural stability. The observed correlations between JRS parameters and LOS outcomes within the FMS group can be attributed to several interconnected factors. Firstly, individuals with better proprioception will likely better perceive their joint angles and body position in space (Proske and Chen, 2021). This heightened awareness enables them to make more precise adjustments in response to postural challenges, ultimately enhancing their ability to maintain stability (Abi Chebel et al., 2022). Secondly, improved proprioceptive abilities may lead to enhanced sensory feedback, allowing individuals with FMS to detect subtle changes in their body position and weight distribution more effectively (Homaye Razavi et al., 2020). This heightened sensory awareness can facilitate quicker and more accurate postural corrections when faced with external disturbances or the need to adapt to varying surfaces (Young, 2020). Thirdly, individuals with better proprioception may exhibit more efficient neuromuscular control, enabling them to activate the appropriate muscles and generate the necessary forces to maintain stability (Salva-Coll et al., 2023). This improved neuromuscular coordination can contribute to their ability to expand their LOS while staying within the limits of their postural control (Faude and Donath, 2019). These findings emphasize the importance of addressing proprioceptive impairments in managing and rehabilitating FMS individuals to potentially enhance their postural stability and overall quality of life (Lim and Park, 2019). However, further research is needed to elucidate the precise mechanisms underlying these observed correlations and develop targeted interventions to capitalize on these relationships to improve outcomes for individuals with FMS.

Neurophysiological and neuroimaging evidence suggests that central sensitization—a heightened responsiveness within the central nervous system—can disrupt afferent proprioceptive processing in FMS, leading to diminished joint position sense and impaired postural control (Quodling et al., 2025). Functional MRI studies have revealed altered activation patterns and reduced gray matter density in sensorimotor cortical regions, including the primary somatosensory cortex, supplementary motor area, and cerebellum, which are essential for proprioceptive integration and balance regulation (Sansare et al., 2025). Additionally, changes in functional connectivity within pain-processing and motor control networks indicate that persistent nociceptive input may reshape cortical body representations, further compromising sensorimotor precision (Kannan et al., 2023). These central alterations, combined with peripheral receptor dysfunction, likely underlie the observed deficits and highlight the need for rehabilitation strategies addressing both peripheral and central mechanisms (Kannan et al., 2023).

This study did not systematically document or statistically control for medication use—particularly analgesics, muscle relaxants, and antidepressants—or for comorbidities such as vestibular disorders, peripheral neuropathy, and cardiovascular disease. Habitual physical activity levels, which may affect proprioceptive acuity and postural stability, were also not quantified. Although exclusion criteria reduced some neurological and musculoskeletal confounders, these unmeasured variables may have influenced the differences in JRS and LOS. Future work should incorporate detailed assessments of medication regimens, comorbidity profiles, and physical activity patterns to more accurately isolate FMS-specific effects.

The FMS group also exhibited higher BMI and lower educational attainment than controls, factors known to influence motor control, sensorimotor integration, and proprioceptive performance. Elevated BMI may alter biomechanical loading and movement strategies, while lower education may reflect differences in health literacy and access to physical activity opportunities. Although not adjusted for in the present analysis, these disparities may have contributed to the observed differences. Future studies should include BMI and education as covariates or examine their effects through stratified analyses.

Psychological factors such as depression, anxiety, and kinesiophobia—common in FMS—were not assessed. These can affect central sensory processing, motor planning, and balance performance through behavioral avoidance and neuromuscular changes. Their omission limits mechanistic interpretation. Future research should integrate validated psychological measures to better understand their contribution and to support development of rehabilitation strategies that address both physical and psychological determinants.

JRS assessments followed a fixed sequence (hip, knee, ankle) to ensure procedural consistency. Although short rest periods were provided and testing was brief, fixed ordering may have introduced chronological effects—positive (learning, familiarization) or negative (fatigue, reduced concentration)—that could influence results. While absent in pilot testing, randomizing joint order in future studies would further reduce this potential bias. Finally, all JRS variables were analyzed in separate regression models to avoid multicollinearity and overfitting given the moderate intercorrelations between measures and the sample size. Although a combined model might reveal interaction effects and relative contributions, such an approach would require a larger cohort to produce stable estimates. Future research should explore integrated modeling to examine joint and interactive influences of multiple proprioceptive measures on postural stability.

Our multiple linear regression analyses shed light on the intricate relationships between JRS in the lower extremities and various facets of postural stability in individuals diagnosed with FMS. Firstly, our findings emphasize the substantial influence of hip JRS on postural stability. Individuals with greater hip joint position errors exhibited longer reaction times, reduced maximum excursion, and diminished direction control (Raizah et al., 2023b). These associations highlight the pivotal role of hip JRS in predicting postural stability outcomes among FM patients (Raizah et al., 2023b). Similarly, our analysis revealed that knee JRS significantly impacted all three postural stability parameters. Higher knee joint position errors were correlated with longer reaction times, decreased maximum excursion, and diminished direction control, underlining the crucial role of knee JRS in predicting postural stability outcomes within the FMS population (Strandkvist et al., 2023). Lastly, our results demonstrated that ankle JRS significantly impacted postural stability. Greater ankle joint position errors were associated with longer reaction times, reduced maximum excursion, and diminished direction control (Strandkvist et al., 2023). These findings collectively underscore the importance of ankle JRS in influencing postural stability among individuals with FM. Overall, our study highlights the intricate interplay between lower extremity JRS and postural stability, providing valuable insights for tailored interventions and treatment strategies aimed at improving the balance and overall quality of life for individuals living with FM (Legendre, 2020).

### Limitations of the study

The cross-sectional design precludes establishing causal relationships between proprioceptive deficits and postural stability impairments. While the regression models identified significant associations, these findings should be interpreted as correlational, and longitudinal or interventional studies are required to determine causal pathways and the temporal sequence of these impairments in FMS.

## Conclusion

In conclusion, this study elucidates the complex relationship between joint JRS and postural stability in individuals with FMS, revealing significant proprioceptive and stability impairments compared with healthy controls. Strong correlations between JRS and LOS indicate that greater proprioceptive accuracy is associated with better postural control. Multiple linear regression analyses confirmed the predictive value of JRS, particularly at the hip, knee, and ankle, for postural stability outcomes. These results support the potential of targeted proprioceptive training to improve stability and functional performance in FMS, warranting further research to optimize rehabilitation strategies and enhance quality of life. Interpretation should consider methodological refinements implemented in this revision, including detailed descriptions of recruitment, screening, and blinding to improve reproducibility. Potential confounders—such as anthropometric, educational, pharmacological, comorbidity, physical activity, and psychological variables—have been acknowledged as possible influences on proprioceptive and postural outcomes, meriting inclusion in future analyses. The discussion also incorporates neurophysiological and neuroimaging evidence to clarify mechanisms underlying observed deficits. Together, these advances provide a stronger foundation for translating biomechanical assessments into targeted, evidence-based rehabilitation strategies for individuals with FMS.

## Data Availability

The datasets presented in this study can be found in online repositories. The names of the repository/repositories and accession number(s) can be found in the article/Supplementary Material.
